# Serological Tests for SARS-CoV-2 Coronavirus by Commercially Available Point-of-Care and Laboratory Diagnostics in Pre-COVID-19 Samples in Japan

**DOI:** 10.3390/diseases8040036

**Published:** 2020-09-23

**Authors:** Zhaoqing Lyu, Mariko Harada Sassa, Tomoko Fujitani, Kouji H. Harada

**Affiliations:** Department of Health and Environmental Sciences, Kyoto University Graduate School of Medicine, Yoshida Konoe, Sakyo, Kyoto 6068501, Japan; lzq_1726362531@yahoo.co.jp (Z.L.); marikohs@kuhp.kyoto-u.ac.jp (M.H.S.); fujitani.tomoko.4w@kyoto-u.ac.jp (T.F.)

**Keywords:** SARS-CoV-2, serological test, LFA, ELISA, specificity, false positive rate, Japan

## Abstract

The number of COVID-19 patients in Japan is considered low, compared with U.S. and European countries. However, recent serological survey reported that several percent of population showed IgG positive to SARS-CoV-2. Specificity in the assays might influence the estimate, and possibility of overdiagnosis should be investigated. Serological tests for SARS-CoV-2 coronavirus were performed in pre-COVID-19 sera in Japan (400 healthy subjects in 2012–2015). Lateral flow assay (LFA) and enzyme-linked immunosorbent assay (ELISA) showed 1.5% (6/400) and 1.75% (7/400) IgG positives, respectively. Among those false positive samples, only one sample was positive in both LFA and ELISA (0.25%; 95% CI: 0.006–1.39%). Possible bias from pooling method was examined by Monte Carlo method and the possibility was unlikely at low false positive rate. Previous surveys might overestimate COVID-19 seroprevalence in several populations of Japan. These false positives could be excluded by combination of different diagnostics. Nonetheless, the result of seroprevalence should be carefully interpreted in less prevalent areas.

## 1. Introduction

The spread of the COVID-19 infection is still not fully understood. Serology is now employed to predict the cumulative incidence of COVID-19 infections [1]. The number of SARS-CoV-2 serological assays such as enzyme-linked immunosorbent assay (ELISA) and lateral flow assay (LFA) is increasing, but external validation has just started. Nonspecific reactions, i.e., cross-reactivity with various common pathogens including other human coronaviruses need to be evaluated [2].

In Japan, the number of COVID-19 patients is still small, but there may be more undiagnosed people. Some preprints and letters have demonstrated several percent seropositivity using LFA [3,4]. However, they were significantly higher than the number of polymerase chain reaction (PCR)-determined symptomatic patients, and effects of false positives are suspected. Therefore, a cross-sectional study using specimens before 2019 from Japanese communities was conducted to evaluate the positive rate (non-specificity) by diagnostics available in Japan.

## 2. Materials and Methods

Cross-sectional serological survey of SARS-CoV-2 antibodies (IgG and IgM) was performed on healthy adults in Kyoto Prefecture, Japan. Participants provided sera at local health check-ups with informed consents, and samples were archived in Kyoto University Environmental Biobank. A total of 400 serum samples from 2012 to 2015 were used for this analysis (80 men, 320 women, average age 63.8 years, standard deviation 13.5 years). LFA (COVI040, GenBody Inc., Cheonan, Republic of Korea) for detecting IgM or IgG and ELISA for IgG (RAI010R, BioVendor Laboratories) against SARS-CoV-2 were used. These diagnostics used the N protein of SARS-CoV-2 as an antigen. Weak signals in LFA were judged as positives. In the ELISA, the lower limit of signal intensity in COVID-19 patients, indicated by the manufacturer, was used as the cutoff value. The analysis was initially conducted for 40 pooled samples from 400 serum samples (10 each by age and sex). Individual sera in pooled samples were examined for the positive pooled groups. If the pooled sera were test-negative, all individual sera in them were considered negative. As a positive control, commercially available convalescent plasma of COVID-19 was used. To further verify reproducibility, individual IgG positive sera in the above LFA and ELISA tests were tested with another LFA (#CG-CoV-IgG, RayBiotech Inc., Peachtree Corners, GA, USA). All three diagnostics were for research use only and are not approved for clinical use in Japan. Monte Carlo simulation was conducted with defined false positive rates (FPR; 0.1%, 0.5%, 1%, 3%, 5%, 10%, 20%) in true non-infected population (400 tests). Dilution effect by pooling was also incorporated in the simulation with assumptions that one, two or three positive samples in a pooled sample would be required for positives in pooled samples. The simulation was repeated 500 times and the distribution of simulated false positive rates were calculated. The Kyoto University Ethics Committee approved this study protocol.

## 3. Results and Discussion

**Figure 1:**
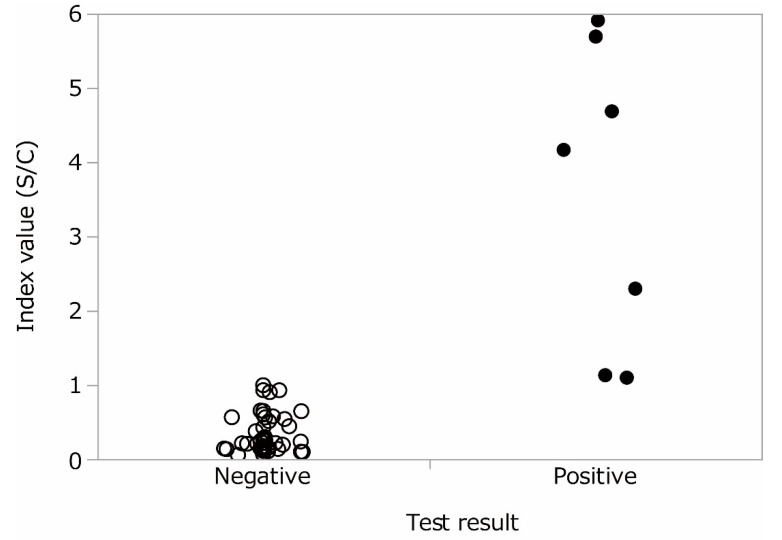
Distribution of signals detected by anti-SARS-CoV-2 NP ELISA in Pre-COVID-19 individual sera in positive pooled samples. Index value, Signal to cut-off (S/C) ratios are plotted.

Positive control samples were tested twice with three different test kits, and all showed positive results. In pre-COVID-19 samples, there were 6 IgG weak positives and 7 IgM weak positives among 40 pooled serum samples in LFA. The individual sera that made up the positive pooled samples were then examined, and 1 IgG positive, 5 IgG weak positives, and 7 IgM weak positives were observed (Table 1). By ELISA, 6 out of 40 pooled serum samples showed IgG positive, and 7 individual samples were IgG positive (1 pooled serum had 2 individual positives). Some other values were seen just below the cutoff value (Figure 1). The same LFA or ELISA tests were performed again on the above positive samples, and a positive reaction were confirmed. From the results, the false positive rates were 1.5% for IgG (95% CI: 0.552–3.24%), 1.75% for IgM (95% CI: 0.706–3.57%) in LFA. In ELISA, it was estimated to be 1.75% (95% CI: 0.706–3.57%). Both LFA and ELISA used in this study showed positive reactions over 1%. The IgM test was usually more nonspecific than IgG test, but IgG also had similar false positive rates. It is likely difficult to estimate the prevalence of COVID-19 by antibody testing in areas where COVID-19 is not prevalent at high rates. In Japan, the number of confirmed COVID-19 infections was 19,282 until 4 July 2020 [5], which was 0.015% of the population of Japan, 125 million. Even when the true seroprevalence of the population was assumed at 0.1% and asymptomatic individuals were tested by an assay with 95% sensitivity and 98.25% specificity, the false discovery rate would be approximately 95%. Reported IgG seroprevalence in several populations in Japan showed around 3–5% [3,4]. However, given that the false positive rate in this study can be applied, those numbers would consist of nonspecific reactions at a high rate.

To evaluate the effect of pooling of samples on the estimate, Monte Carlo method was used to simulate the possible bias between individual assays and pooled assays (Table 2). At lower false positive rates (less than 3%), deviation in pooled assays with highest detection sensitivity showed less than 10% to individual assays. Particularly at 1% FPR (observed in this study), ‘Pool 1’ (1.0% (95% CI 0.3–1.8%)) demonstrated similar performance to individual assays (1.0% (95% CI 0.3–2.0%)). If the pooling method caused lowering of sensitivity, positive reactions would drastically decrease, for example at 1%, 0% (0–0.3%) in ‘Pool 2’ scenario. Indeed, in this study, most of individual assays of samples in positive pooled samples showed single positive reaction (except for one of 13 positive pooled sample). Hence, the pooling method in this study would not affect the validity of the estimate.

Monte Carlo simulation was conducted with defined FPR (0.1, 0.5, 1, 3, 5, 10, 20%) for 400 tests. ‘Individual’ means rates of positives in individual 400 tests. ‘Pool 1’ means rates of positives in 40 pooled samples that were identified as positive when at least one sample in a pool was positive. ‘Pool 2’ and ‘Pool 3’ mean rates of positives in 40 pooled samples that were identified as positive when more than one or two sample in a pool were positive, respectively.

These positive cases were partially overlapped between tests. Only one case showed positive for both IgG and IgM in LFA. Another one patient showed positive by both LFA and ELISA (0.25%; 95%CI: 0.006–1.39%) (Table 1). A total of 12 IgG positive samples were further assayed with additional LFA, and weak signals were detected in only 1 of 6 LFA IgG positives and 2 of 7 ELISA IgG positives. None of the samples tested positive in all three tests. Specificities can vary among researches and diagnostics due to qualitative readings, cutoff values, etc. Nevertheless, in this study, there was almost no agreements in different test kits for false positives, then it is possible to reduce the false positives by using multiple diagnostics for positive samples. Even then, whether the specificity of 99.75% is sufficient or not, should be taken into consideration in the epidemic situation. On the other hand, although the effect on sensitivity has not been examined in this study, it will also be a trade-off relationship with specificity.

## Figures and Tables

**Table 1:** Comparison of anti-SARS-CoV-2-NP IgG and IgM assay specificities in LFA and ELISA in Pre-COVID-19 sera.

**(A) Test Results for IgG between LFA and ELISA**			
no. (%)	LFA (+)	LFA (−)	Total
ELISA (+)	1 (0.25%)	6 (1.5%)	7 (1.75%)
ELISA (−)	5 (1.25%)	388 (97%)	393 (98.25%)
Total	6 (1.5%)	394 (98.5%)	400
**(B) Test Results between IgG and IgM in LFA**			
no. (%)	IgG (+)	IgG (−)	Total
IgM (+)	1 (0.25%)	6 (1.5%)	7 (1.75%)
IgM (−)	5 (1.25%)	388 (97%)	393 (98.25%)
Total	6 (1.5%)	394 (98.5%)	400

LFA: lateral flow assay, ELISA: enzyme-linked immunosorbent assay, (+): positive, (−): negative; IgG: immunoglobulin G, IgM: Immunoglobulin M.

**Table 2:** Mote Carlo simulation of false positives in pooled samples with different false positive rates (FPR) and detection sensitivity.

FPR	0.1%	0.5%	1%	3%	5%	10%	20%
Individual	0.0%	0.5%	1.0%	3.0%	4.8%	9.8%	20.0%
	(0.0–0.5%)	(0.0–1.3%)	(0.3–2.0%)	(1.8–4.5%)	(3.3–7.0%)	(7.5–12.5%)	(16.8–23.5%)
Pool 1	0.0%	0.5%	1.0%	2.8%	4.0%	6.5%	9.0%
	(0.0–0.5%)	(0.0–1.3%)	(0.3–1.8%)	(1.5–3.8%)	(2.8–5.4%)	(5.1–7.8%)	(8.0–9.8%)
Pool 2	0.0%	0.0%	0.0%	0.3%	0.8%	2.5%	6.3%
	(0.0–0.0%)	(0.0–0.0%)	(0.0–0.3%)	(0.0–0.8%)	(0.3–1.8%)	(1.5–3.8%)	(5.0–7.8%)
Pool 3	0.0%	0.0%	0.0%	0.0%	0.0%	0.8%	3.3%
	(0.0–0.0%)	(0.0–0.0%)	(0.0–0.0%)	(0.0–0.3%)	(0.0–0.5%)	(0.3–1.5%)	(2.0–4.5%)

Results showed median and 95% intervals of simulated results of 500 iterations.
